# Diogenes Syndrome: A Case Report

**DOI:** 10.1155/2013/595192

**Published:** 2013-01-31

**Authors:** Projna Biswas, Anusree Ganguly, Sanchaita Bala, Falguni Nag, Nidhi Choudhary, Sumit Sen

**Affiliations:** Department of Dermatology, IPGMER and SSKM Hospital, 244 A. J. C. Bose Road, Kolkata, West Bengal 700020, India

## Abstract

Cessation of normal skin cleansing seen in geriatric or self-neglected patients can cause accumulation of keratinous crusts on the skin. In the extreme end of this spectrum is a condition known as Diogenes syndrome (DS). These patients may have psychiatric disorders like paranoid disorders, mood affection, or temporofrontal dementia. Subjects are mainly the elderly but few cases in younger age group of patients have also been reported. Lesions of DS are usually found over upper central chest, back, and groin. In the young, lesions are mainly found over scalp, face, or arms. Absence of normal skin cleaning causes keratin and dirty debris to accumulate and with time form a thick shell. These debris can be secondarily infected by bacteria, fungus, and so forth. These skin lesions are not usually seen in individual with proper hygiene. We report a case of Diogenes syndrome in a 34-year-old young male patient who had associated schizophrenia.

## 1. Introduction

Diogenes syndrome (DS) is characterized by self-neglect and social withdrawal with abnormal collecting pattern, tendency to hoard excessively (syllogomania), and refusal of help, wich may be precipitated by stressful event [1]. DS is named after the Greek Philosopher “Diogenes of Sinope” (4th century BC) who taught about cynicism philosophy. He kept his need for clothing and food to a minimum by begging. He used to follow some ideas like “life according to nature,” “self-sufficiency,” “freedom from emotion,” “lack of shame,” “outspokenness,” and “contempt for social organization” [2]. Diogenes syndrome has been described in different psychiatric literature and very few cases associated with dermatological presentation have been reported. 

## 2. Case Report

**Figure 1:**
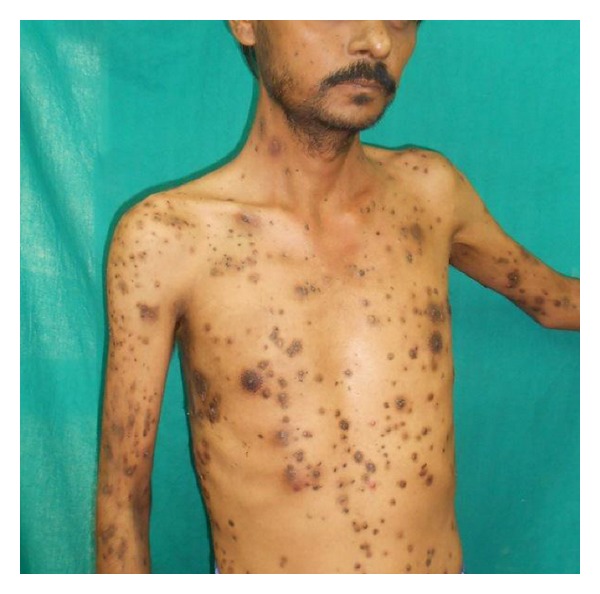
Crusted lesions on erythematous base over the trunk.

**Figure 2:**
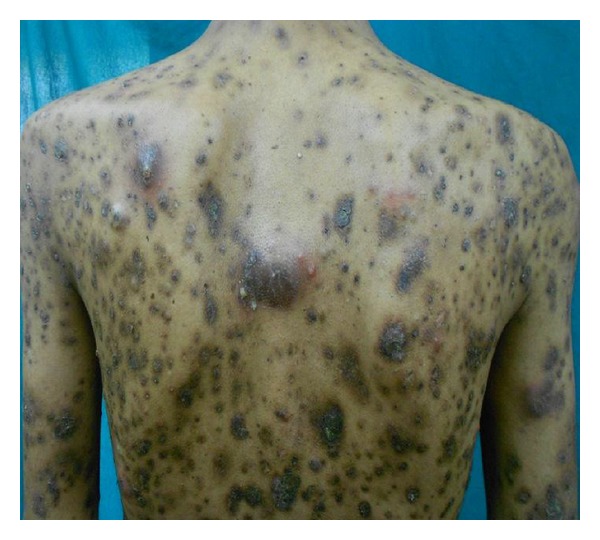
Multiple furuncles and acneiform lesions over back.

**Figure 3:**
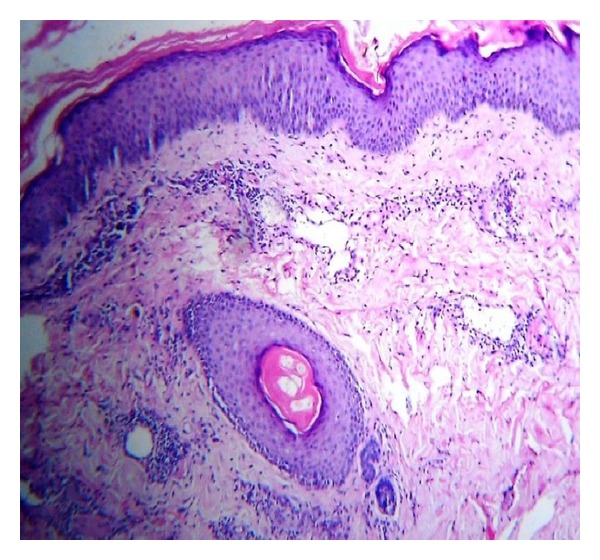
Hyperkeratosis and upper dermal infiltrate. (H&E: ×100).

**Figure 4:**
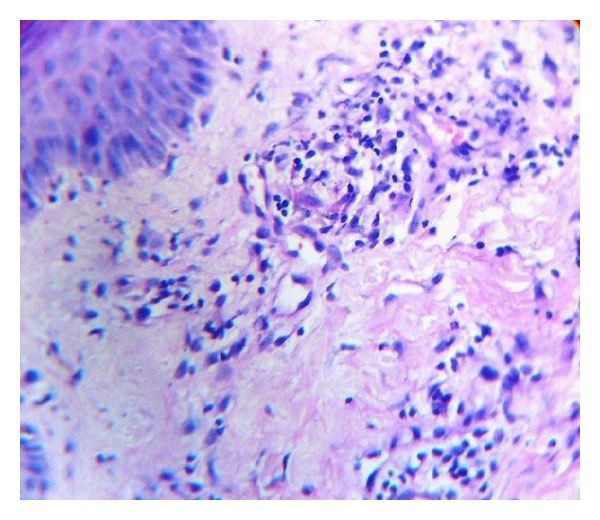
Perivascular and periappendageal infiltration of neutrophils, lymphocytes, and macrophages. (H&E: ×400).

A 34-year-old male patient was brought to our OPD by his neighbours with multiple discrete papulonodular lesions mainly over the trunk with heaped up crusting, for the last 6 months. According to the persons who coaxed him to come to the OPD, the patient lived alone with least interaction with his neighbours and had no relatives to visit him. They also reported that the man had not taken a bath for longer than 2 years. His home was filthy and was crowded with furniture, old books, and scraps of papers in huge heaps. On examination multiple nodular lesions with crusting on an erythematous base were present mainly over the trunk and upper extremities (Figure 1). Few lesions were acneiform and there were multiple furuncles (Figure 2). His scalp, groin, and face were almost spared. There was no suggestive feature of scabies or pediculosis clinically. Patient was alert and cooperative but was severely depressed and had been stubbornly refusing help from neighbours. He was a graduate and working as a computer programmer. We sent the patient to the psychiatry OPD and there he was diagnosed as a case of schizophrenia. Skin biopsy was taken from a lesion and under light microscopy it showed hyperkeratosis with mainly upper dermal infiltrate. Inflammatory cells were mostly found at perivascular and periappendageal regions and consisted of plenty of neutrophils with few lymphocytes and macrophages (Figures 3 and 4). Routine blood investigation and X-ray chest was noncontributory. His blood VDRL in titre were nonreactive. We also did Gram staining, bacterial and mycological culture from the discharge of the lesion, which did not reveal growth of any organism. With regular washing and treatment with antibiotics and antipsychotics improvement was satisfactory. 

## 3. Discussion

Diogenes syndrome is also known as dermatitis passivata. The term Diogenes syndrome was coined in 1975 by “Clark et al.” [3]. This usually affects elderly persons and there is no sex predilection. The disorder follows a distinct sociodemographic profile where it is found that persons are usually single, aged, having average or above average intelligence, and also having good income [1]. DS has been classified as primary or pure which is not associated with mental illness and secondary or symptomatic. Secondary DS is related to mental illness like schizophrenia, depression, and dementia [1]. Alcohol abuse has been identified as a cofactor [4]. Certain characteristics of the Diogenes syndrome have been recognized; these include social withdrawal, filthy home, neglected self care, squalor syndrome, collection of useless objects or hoarding, shameless attitude, and stubborn refusal of help [1]. At least 4 of them are almost permanent symptoms: patients do not ask for any help although they possesses nothing; unusually fond of objects (hoarding of rubbish, or nothing in the house); unusual behavior with other people (misanthropy) and extreme self-neglect [5]. Hoarding may be absent in some cases [6]. A question has been raised whether DS is due to self-neglect or maltreatment of the elderly but the latter has been identified as the most probable cause resulting in this pitiful condition [1]. Though principally affecting the elderly, young persons have been diagnosed with this condition. Such persons usually have above average intelligence and it is now clear that some stressful event precipitates the disease in predisposed individuals. Multiple deficiency states have been associated with DS including iron, folate, vitamin B12, vitamin C, calcium and vitamin D, serum proteins and albumin, water, and potassium [3].

Skin lesions are mainly due to uncleanliness which may result in various infestations and infections. These are ignored by the patient. Dirt, dust, bacterial, fungal, and parasitic debris conglomerate to form thick crusts and scales over various parts of the body [4].

The case has been reported to increase medical awareness and to highlight that young individuals living in self-imposed isolation may suffer from this psychological impairment with suggestive cutaneous manifestations.
